# Probiotics for Neurodegenerative Diseases: A Systemic Review

**DOI:** 10.3390/microorganisms11041083

**Published:** 2023-04-20

**Authors:** Sandhya Ojha, Nil Patil, Mukul Jain, Chittaranjan Kole, Prashant Kaushik

**Affiliations:** 1 Cell & Developmental Biology Laboratory, Centre of Research for Development, Parul University, Vadodara 391760, India; san15720@gmail.com (S.O.); patilnil910@gmail.com (N.P.); 2 Department of Life Sciences, Parul Institute of Applied Sciences, Parul University, Vadodara 391760, India; 3 The Neotia University, Sarisha 743368, India; 4 Instituto de Conservacióny Mejora de la Agrodiversidad Valenciana, Universitat Politècnica de València, 46022 Valencia, Spain

**Keywords:** probiotics, neurodegenerative diseases, gut microbiota, aging, systematic review

## Abstract

Neurodegenerative disorders (ND) are a group of conditions that affect the neurons in the brain and spinal cord, leading to their degeneration and eventually causing the loss of function in the affected areas. These disorders can be caused by a range of factors, including genetics, environmental factors, and lifestyle choices. Major pathological signs of these diseases are protein misfolding, proteosomal dysfunction, aggregation, inadequate degradation, oxidative stress, free radical formation, mitochondrial dysfunctions, impaired bioenergetics, DNA damage, fragmentation of Golgi apparatus neurons, disruption of axonal transport, dysfunction of neurotrophins (NTFs), neuroinflammatory or neuroimmune processes, and neurohumoral symptoms. According to recent studies, defects or imbalances in gut microbiota can directly lead to neurological disorders through the gut-brain axis. Probiotics in ND are recommended to prevent cognitive dysfunction, which is a major symptom of these diseases. Many in vivo and clinical trials have revealed that probiotics (*Lactobacillus acidophilus*, *Bifidobacterium bifidum*, and *Lactobacillus casei*, etc.) are effective candidates against the progression of ND. It has been proven that the inflammatory process and oxidative stress can be modulated by modifying the gut microbiota with the help of probiotics. As a result, this study provides an overview of the available data, bacterial variety, gut-brain axis defects, and probiotics’ mode of action in averting ND. A literature search on particular sites, including PubMed, Nature, and Springer Link, has identified articles that might be pertinent to this subject. The search contains the following few groups of terms: (1) Neurodegenerative disorders and Probiotics OR (2) Probiotics and Neurodegenerative disorders. The outcomes of this study aid in elucidating the relationship between the effects of probiotics on different neurodegenerative disorders. This systematic review will assist in discovering new treatments in the future, as probiotics are generally safe and cause mild side effects in some cases in the human body.

## 1. Introduction

Neurodegenerative diseases are a group of disorders characterized by the progressive loss of structure and function of neurons in the central and/or peripheral nervous system [1]. These diseases affect various parts of the brain, spinal cord, and nerves, leading to a gradual decline in cognitive, motor, and/or sensory function [2]. There is emerging evidence to suggest that probiotics, which are live microorganisms that provide health benefits when consumed in adequate amounts, may have a role to play in the prevention and treatment of neurodegenerative diseases [3]. Studies regarding probiotics suggest that utilization of probiotics increased intestinal flora which can regulate the inflammatory response, as well as resist the colonization of exogenous pathogenic microorganisms [4].

While probiotics are generally considered safe and can provide numerous health benefits, there are several limitations to their use. Here are some of the most important ones:

(1) Limited evidence for specific health conditions: While probiotics have been shown to be effective in treating certain conditions, such as diarrhea caused by antibiotics, there is limited evidence to support their use in treating other health conditions. For example, while some studies have suggested that probiotics may be beneficial for conditions such as irritable bowel syndrome (IBS) and eczema, the evidence is not yet strong enough to make definitive recommendations [5]. (2) Variability in strains and dosages: Different strains of probiotics can have different effects on the body, and the appropriate dosage can vary depending on the specific strain being used. Therefore, it can be difficult to determine the optimal strain and dosage for a particular health condition, and the effectiveness of probiotics can vary widely depending on these factors [6]. (3) Limited survival and colonization: Probiotic bacteria must survive the acidic environment of the stomach and colonize the gut in order to provide health benefits. However, not all probiotic strains are able to survive this process, and even those that do may have difficulty colonizing the gut in sufficient numbers to have a meaningful effect [7]. (4) Short-term effects: Probiotics are typically used for short periods of time, often a few weeks or months. However, the long-term effects of probiotic use are not well understood, and it is possible that their benefits may diminish over time [7]. (5) Potential adverse effects: While probiotics are generally considered safe, they can sometimes cause adverse effects such as bloating, gas, and diarrhea. In rare cases, they may even cause serious infections, particularly in people with weakened immune systems [8]. However, contrary to its side effects, it is highly effective, and healthy gut microbiota can help reduce inflammation and oxidative stress, which are two key factors that contribute to the development of neurodegenerative diseases [9]. Additionally, some probiotics have been shown to create molecules that can stimulate the production of brain-derived neurotrophic factor (BDNF), a protein that promotes the growth and survival of neurons in the brain. Low levels of BDNF have been linked to several neurodegenerative diseases, including Alzheimer’s disease and Parkinson’s disease [10]. While the research on probiotics and neurodegenerative diseases is still in its early stages, there is growing interest in exploring the potential benefits of probiotics in this area. However, more research is needed to determine the optimal strains, dosages, and treatment regimens for different neurodegenerative diseases.

A systematic review is a type of research study that collects and analyzes all available evidence on a specific topic, in order to answer a specific research question [11]. In the case of probiotics and neurodegenerative diseases, a systematic review can help to provide a comprehensive overview of the current state of research on this topic. There are several reasons why a systematic review is important for examining the effects of probiotics on neurodegenerative diseases. Firstly, there is a large and growing body of research on this topic, and a systematic review can help to synthesize this information and identify the most promising areas for future research. Secondly, neurodegenerative diseases are complex and multifactorial disorders, and it is likely that probiotics may have different effects on different aspects of these diseases. For example, probiotics may have different effects on cognitive function, motor symptoms, and inflammation in different neurodegenerative diseases [12]. A systematic review can help to identify these differences and provide guidance on the most appropriate use of probiotics for different patient populations. Thirdly, there is currently a lack of consensus on the optimal probiotic strains and dosages for different neurodegenerative diseases. A systematic review can help to identify the most effective probiotic interventions and inform the development of future clinical trials. In summary, a systematic review is an important tool for evaluating the effects of probiotics on neurodegenerative diseases, as it can provide a comprehensive and evidence-based overview of the current state of research on this topic.

## 2. Methods

### 2.1. Literature Search Strategy

The recommended notification items for systematic reviews and meta-analyses (PRISMA) recommendations are used to direct the literature search [13]. Three electronic databases were searched from inception to March 2023: MEDLINE/PubMed, Springer Link, and Nature with Probiotics and Neurodegenerative diseases keywords. No publication year restrictions were applied and only English literatures and Free open access literature were used.

### 2.2. Inclusion and Exclusion Criteria

Inclusion and Exclusion criteria used in this systematic study is described in below Table 1.

### 2.3. Management of Extracted Data

**Figure 1:**
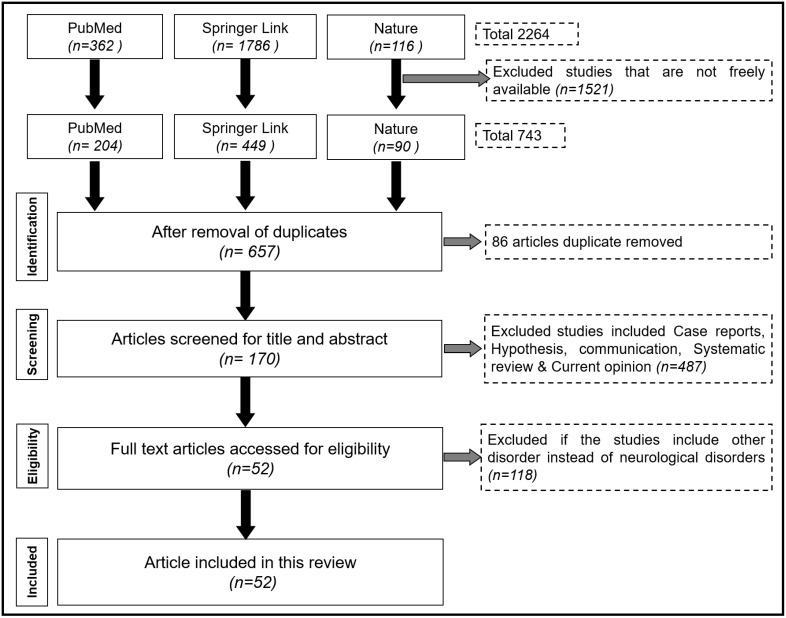
Identification and screening for literature search.

Literature screening is based on Figure 1. Each article’s abstract and title are first separately reviewed by two evaluators. (N.P. and S.O.). Each of the papers that were chosen had to have covered one of the following metrics that were mentioned in the selection criteria. Any discrepancy is resolved with discussion and consultation of third reviewer (M.J. and P.K.). No replication or dispersion data are mentioned for in vivo experiments. In vivo studies are found to be free of bias, in contrast to clinical trials, which are subject to a bias evaluation. We assure that there is no duplication of any literature.

### 2.4. Strategy of Data Extraction

The data extraction provides the results of the chosen papers describe in tabular format in Section 4.6, while the study’s findings were discussed in Section 3. The Section 4 explained how the results were analyzed.

### 2.5. Data Synthesis and Statistical Analysis

If there was heterogeneity between articles, data were combined using a random effects model to provide a more cautious estimate of the spectrum of probiotics in neurodegenerative diseases [14]. Both the *I*^2^ index 50% and the chi-squared test with a *p* < 0.05, used to identify a significant degree of heterogeneity, were used to evaluate heterogeneity, which is variance between individual research findings that has not occurred by accident [15]. All statistical analysis was conducted through MS Excel (Windows XPC) by reviewer M.J.

## 3. Results

### 3.1. Literature Search and Selection

An initial search of the literature identified 2264 potentially related articles. Due to the free access of articles, 1521 were excluded from the study. The remaining 743 articles were further evaluated on the basis of duplication and 86 articles were removed. Around 487 articles are removed as they are under the exclusion criteria of Case reports, Hypothesis, Communication, Systematic review and Current opinion. The final 52 articles are full texts and eligible for the systematic review and discussion.

### 3.2. Characteristics of the Included Studies

We identified 20 articles regarding Parkinson’s disease and 32 Articles regarding Alzheimer’s disease with probiotics that have reported the potential effect of probiotics in neurodegenerative diseases. Table 2 describes the selected studies according to the type of probiotics, their effects, and their mechanism of action in particular neurodegenerative diseases.

## 4. Discussion

### 4.1. Gut Microbiota-Brain Axis

**Figure 2:**
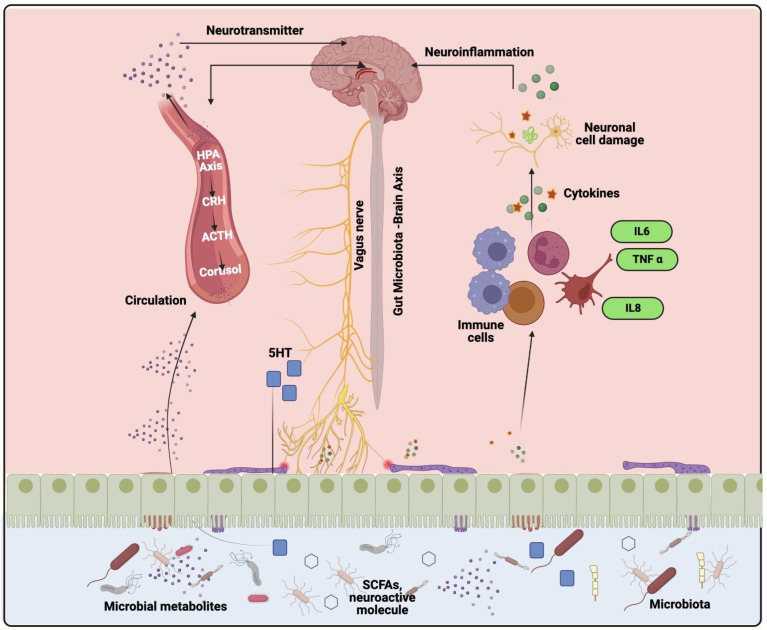
Microbiota–gut–brain axis. Through neural, metabolic, endocrine, and immunological mechanisms, the brain and stomach interact. The vagus nerve, the hypothalamic-pituitary-adrenal (HPA) axis, and systemic circulation are all influenced by the brain’s function on gastrointestinal health. Short-chain fatty acids (SCFAs), neurotransmitters, and amino acids are examples of signals from the stomach that modify brain activity through neuronal cells, the immune system, and hormonal processes.

The gut-brain axis is a complicated biochemical pathway that connects the gastrointestinal tract and the brain, it is a bi-directional interaction between GI and CNS microbiota via endocrine, neural, humoral, and immune links Figure 2 [16].

This communication is responsible for both healthy and unhealthy benefits. The diversity of gut microbiota has both pathogenic and commensal roles, such as *Lactobacillus* releasing short-chain fatty acids and acetylcholine. Whereas Bacillus produced norepinephrine and dopamine [17]. The gut microbiota performed some functions repeatedly in a cyclic way in the brain such as regulation of the hypothalamic-pituitary-adrenal axis, which released cortisol that is going to activate the brain microglia and releases cytokines. They also control the PNS and CNS by establishing a connection between vagal nerves, adrenergic nerve activation, and several immunomodulatory, neuropeptides, endocrine hormones, and neurotransmitters. Gut microbiota diversity and their action can be affected by hormones such as noradrenaline [18]. If the gut epithelial barrier is being dysregulated, it disturbed the brain-gut-microbiota axis, which encourages the production of neuroactive substances and neurotropic viruses that regulates pathogens with slow neurotoxic properties [19]. The immune responses of the gut are harmonized by the microbial community present in the gut by various cells that maintain various immunological conditions. Microbiota of the gut can be analyzed by Next-generation sequencing and meta-genome analysis reveals that the mammalian gut has microbiomes (most commonly *Cryptosporidium* sp. *Shigella* sp., *Enterotoxigenic Escherichia coli*) [20]. Bacteria are hardly able to pass the blood-brain-barrier or blood-cerebrospinal fluid barrier to enter CNS, by the mechanism called trans-cellular infiltration, infected leukocytes, and para-cellular entering [21]. The mechanism that may influence neurodegeneration through gut microbiota, is the production of functional metabolites transferred through vagus nerves, microbial-associated molecular patterns, and unable to inhibit harmful gut microbes. The pathological process of the gut microbiome is to release neurotoxic metabolites that can be transferred through the gut-brain axis or microbial-molecular patterns, and are unstoppable by the peripheral immune system [22].

### 4.2. Relation of Gut Microbiota with Neurodegenerative Disorders

The health of the brain is synchronized or regulated by the GI tract or it is directly proportional to the microbiome present in the human gut. Imbalance in the microbial community is associated with many diseases, but in the brain, it was responsible for neurodegenerative diseases such as AD, PD, and others disorders [23]. Brain and gut microbiota can interrelate with each other through several pathways such as neuroendocrine, neuroimmune, and autonomic nervous systems. Interactive partners that perform the mechanisms are the cell wall, neurotransmitters, vagus nerves, and metabolites [24]. The microbiome of the gut can synthesize the neurotransmitter that may help to maintain the homeostasis of the central nervous system, which can influence neurodegeneration, examples are tryptophan, brain-derived neurotrophic factor (BDNF), Gama-aminobutyric acid (GABA), and short-chain fatty acid (SCFA) [25]. Neurotrophins have a neuroprotective property that is important for the growth, development, and synaptic plasticity along with the differentiation and survival of the neuron. The decreased level of BDNF influences the neurodegenerative disease related to the cerebral cortex and directly relates to the gut-brain axis, which triggers other diseases too [26]. Gut microbes such as *Faecalibacterium prausnitzil*, *Clostridium leptum* and *Eubacterium rectala*, etc. produce short-chain fatty acids through a down regulation of pro-inflammatory cytokines that play a major role in neurodegeneration. Microbial-derived SCFAs are produced by bacterial fermentation and have neuro-active functions, they act as a modulator for serotonin (neurotransmitter) and some neuropeptides which help to facilitate the gut-brain axis at various stages. During the release mechanism which influences neuronal health and behavioral response [27]. Excessive release of SCFAs is responsible for neuronal health and behavioral responses. An essential amino acid called tryptophan plays an important role in the synthesis of serotonin and other neurotransmitters in the CNS. Imbalance in their levels leads to brain and gastrointestinal disturbances that may cause neurodegeneration, cognitive impairment, and mood disorders [28,29]. An important inducer that administered the excitation of the neurons, is a by-product of bacterial metabolism called GABA. Deregulation of GABA leads to various pathological imbalances that play a major role in neuro-cytotoxicity which accelerates several chronic neurological disorders. GABA is an example that proves how gut microbiota regulates brain chemistry [30].

#### 4.2.1. Alzheimer’s Disease (AD)

People with gut disorders more prone to have AD in the future. Changes in complex ecosystems are co-related with many gastrointestinal disturbances that can implicate many inflammatory diseases including obesity, diabetes and inflammatory bowel disease. The dysbiosis of gut microbiota has an impact on the synthesis of proteins and metabolic processes which are related to the progression of the disease such as AD. Aging changes the gut microbial concentration which enhances pro-inflammatory bacterial growth more than anti-inflammatory bacteria that deteriorates the permeability of the blood-brain barrier and GIT (Gastro Intestinal Tract) functions [31]. Pro-inflammatory phylum such as *Proteobacteria*, *Verrucomicrobia*, genera such as *Escherichia/Shigella*, and species such as *Pseudomonas aeruginosa*, anti-inflammatory species are *Clostridium* spp., and *Ruminococcaceae*. Some study reveals that increased mRNA encoding initiates the release of pro-inflammatory cytokines such as, IL6, CXCL2, and NLRP3, *Escherichia/Shigella* are related to pro-inflammatory taxon [28]. The presence of *Helicobacter pylori* in the gut microbiota increases the release of inflammatory mediators which increases the amyloid β 40/42 ratio in the serum, other bacteria such as *Borrelia burgdorferi* and *Chlamydia pneumoniae* also participate in the hyper phosphorylation of tau which is an important hallmark of AD. IL-10 is an anti-inflammatory cytokine *Eubacterium rectale* is associated with an anti-inflammatory taxon [32].

#### 4.2.2. Parkinson’s Disease

PD is a multifocal neuronal disease that is distinguished by tremors, slow movement, akinesia, muscular rigidity, gait, and difficulty in walking. Instead of these symptoms, PD patients suffer from constipation which is one of the causes of increased intestinal permeability and inflammation that is directly related to the microbiota community of the small intestine [33]. Small intestinal bacterial outgrowth and helicobacter pylori infection has seen in diseased person that causes motor impairment and problem-related with stool [34]. In most cases, patients suffer from increased mucosal permeability and endo-toxic exposure caused by Coliform bacteria [35]. Comparably, some bacteria are reduced in feces such as *Roseburia intestinalis*, *Roseburia hominis*, *Coprococcus eutactus*, and *Blautia faecis* dysregulate the biosynthesis of lipopolysaccharides and are also responsible for GABA deregulation [36]. *Escherichia coli* is a Gram-negative bacteria that releases amyloidogenic protein which induces alpha-synuclein aggregation and which regulates disease in the gut and neurodegeneration in the brain [37].

#### 4.2.3. Huntington’s Disease

HD is a genetic disease caused by overexpression of the huntingtin coding gene, new research suggests that an imbalance in gut microbiota dysregulates the cytokine levels and excessive production of hydrogen sulfide that negatively affects gut health [38,39]. Imbalance is seen in two microbial communities such as increased the majority of the bacterial phyla *Bacteroidetes* (4%) and *Firmicutes* (83%) which causes mortality and motor ability-related problems in HD conditions. Research suggests that irregular intestinal biomicrome decreases mucosal thickness and decreased neuropeptide formation with abnormal endocrine hormonal conditions [40,41,42]. ATP levels are associated with *Prevotella scopos* which has negative effects and also has a co-relation with decreased butyrate formation affected by *Blautia producta* [43].

#### 4.2.4. Other Neurological Disorders

In neurological disorders, the nerve function and structure become affected badly, causes of neuronal cell death, are Amyotrophic lateral sclerosis, Friedreich ataxia, Lewy body disease, spinal muscular atrophy, and Epilepsy, etc. Epilepsy, a neuro-psychiatric disorder is a result of environmental and genetic imbalance, several studies implicated dysbiosis of gut microbiota correlated with the disease, and imbalance of microbiome increases the pro, and anti-inflammatory effects, which leads to chronic inflammation and progression of the disease [44,45]. Bacteria out-growth downregulates lipid and glucose metabolism which disturbs the ATP binding cassette and transporter-associated pathways [46]. Amyotrophic lateral sclerosis is a degenerative condition that is caused by the mutation in dozens of genes which produces a misfolded protein that is found in motor neurons, responsible for voluntary muscular movement [47]. Bulbar function slowly deteriorated with the progression of the disease, and dysphagia (because of aspiration pneumonia and weight loss) have seen [48]. ALS is implicated by the deregulation of the resident and peripheral immune system. Gut microbiota connected with the intestinal immune system, because of invasion or dysbiotic leaky gut and disturbed molecular patterns, provoke cells to release pro-inflammatory cytokines that deregulate the *Firmicutes*/*Bacteroidetes* ratio [49]. Bacteroidetes are good gut bacteria, that are decreasing at the diseased condition that imbalances the cell homeostasis, is regarded as dysbiosis and several reviews suggest that the gut plays an impotent role in the progression of Lewy body disease, and intestinal pro-inflammation is the cause of the chronic phase of the disease. An imbalance in microbiota and translocation of the lumen through a leaky gut are key mechanisms of the disease [50].

### 4.3. Elucidation the Role of Gut Microbiota in Neuroinflammation

Activation of immune cells defined as a pro-inflammatory condition, is an important pathophysiological aspect, behind all neurodegenerative disorders, and leads to neuroinflammation, mediated by the secretion of chemokine, cytokine, ROS, and secondary messengers [51]. CNS is protected from all kinds of toxins through the blood-brain barrier, and its disruptive permeability accumulates neurotoxin waste that increases immune cell influxes [52]. Colonization of *Helicobacter pylori* is responsible for dysbiosis in the gut and causes systemic inflammation that accelerates pathologies for neurodegeneration and other disorder such as asthma, metabolic syndrome, and allergy. The phyla that constitute this core microbiota are Bacteroides and Firmicutes, which are the dominant phylum, in addition to *Lentisphaerota*, *Actinomycetota*, *Spirochaetota*, *Verrucomicrobiota*, *Proteobacteria*, and *Fusobacteriota*. Microbiomes produce metabolites involved in cell homeostasis such as SCFAs regulate the homeostasis of microglia, and induced T cell activation to fight autoimmunity [53]. Tryptophan metabolism facilitates the CNS-microbiome interaction through metabolic by-products such as indole tryptamine, indole acetic acid, quinolinate, and indole propionic acid are neuroactive metabolites and able to regulate CNS activity [54]. These metabolites enter in CNS through the leaky gut epithelium and alteration in Blood-Brain-Barrier facilitates the endotoxins to enter into CNS. Vegas nerves, play an important role to define CNS-microbiome relations, are directly connected with the gut and brain, and are also able to sense bacterial metabolites by their afferent composition. Vegas nerve transfers this information, which has concerns about altered microbiota to CNS, so that appropriate action should be initiated to avoid neuropathological conditions [55]. Blood-brain-barrier designed to protect CNS from the toxin and pathogens that can damage the parenchymal lining and disrupts its working which allows the entry of neurotoxins and microbial metabolic wastes into the CNS, which can be one of the causes of neuroinflammation [56].

### 4.4. Understanding the Role of Probiotics in Gut Microbiota

**Figure 3:**
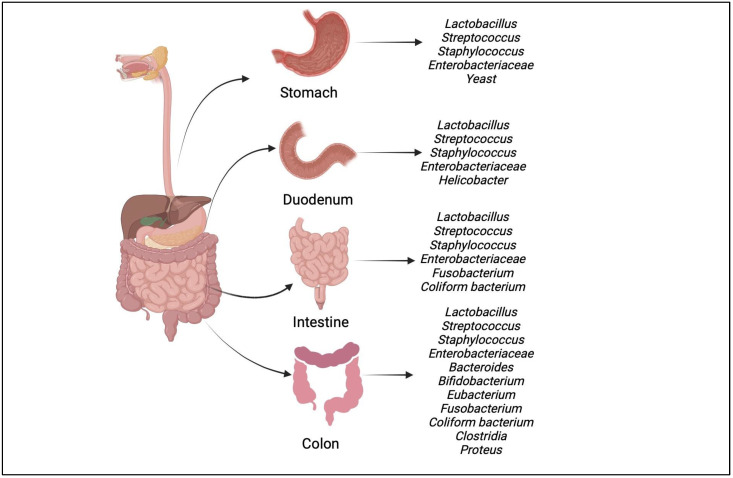
The composition of the microbiome varies with the position of the organ in the human body. This figure shows promising bacterial genera in the stomach, colon, and small intestine.

Probiotics were first proposed by Nobel Prize recipient Elie Metchnikoff at the turn of the 20th century. According to today’s meaning, probiotics are living microorganisms that, when given to a host in sufficient quantities, help their health Figure 3 [57]. The management of gut microbial communities, the repression of pathogens, immunomodulation, promotion of epithelial cell proliferation and differentiation, and reinforcement of the digestive barrier are some of the mechanisms of probiosis [58]. The connections between gut microbes and the immune system have revealed previously unknown microbial components or receptors that, in addition to the conventional immune components, also regulate energy, glucose, and lipid metabolism, in reference to that Table 2.

Cani et al., 2007 introduced the idea of metabolic endotoxemia. Notably, a slight increase in blood LPS was found to be a crucial factor in the onset of low-grade inflammation, and eventually insulin resistance in models of genetically predisposed or diet-induced obesity and diabetes, as well as related cardio metabolic diseases [59]. The relevant and significant literature concentrates mainly on different strains, from more recent prospects such as *A. muciniphila* and *Faecalibacterium prausnitzii* are considered to be superior to more traditional probiotics such as *Lactobacillus* and *Bifidobacterium* or the yeast *Saccharomyces boulardii* [60]. According to research by Bo et al., 2020, *Bifidobacterium pseudolongum* was able to reverse the dysbiosis of the gut microbiota in obese mice, including the variety of the microbiome and the proportion of Firmicutes to Bacteroidetes [61]. The bacterial species *Bifidobacterium* and *Butyricimonas* were also more prevalent after this therapy. Furthermore, the defense system has an impact on the central nervous system (CNS). In light of this, immunity has a direct impact on people’s quality of life, and controlling gut bacterial populations with probiotics has proven to be a successful strategy for doing so [62]. This systematic study reviews the impact of probiotics on human immunity and the gut microbiome in relation to neurodegenerative diseases (mainly AD, PD, and HD).

**Table 2:** Popular probiotic strains, their location in the gut, function in the human body, and health benefits with their source (food).

Strain Name	Location in Gut	Function	Source (Food)	Health Benefits	Refs.
*Bifidobacterium bifidum*	Interior intestine	Activate the host immunity, adhere to gut mucosa, and metabolize host glycan (mucin)	Yogurt, Kefir, Sauerkraut, Garlic	Prevent inflammation, Enteric cancer, Ulcerative colitis and depression	[63,64]
*Bifidobacterium breve*	Gastrointestinal tract	Modulating expression of inflammatory receptors	Kombucha, water kefir, and raw sauerkraut	Prevent pediatrics, For pathologies such as diarrhea and infant colics, to celiac disease, obesity, allergic and neurological disorders	[65,66,67]
*Bifidobacterium longum*	Interior intestine	Inhibits inflammation by regulating the immune system, improving the intestinal barrier function, and increasing acetate production	Goat dairy products, such as yogurt, kefir, seaweed, and miso soup	Reduced stress and improved memory, improving irritable bowel syndrome	[68,69,70]
*Bifidobacterium animalis*	Majorly in animal intestine	Reduce the inflammatory receptor expression	Mammalian colon and Milk	Constipation, irritable bowel syndrome (IBS), respiratory system infections, and excessive screaming in infants	[71,72]
*Bifidobacterium catenulatum*	On the wall of GI	Maintain functional integrity of gut	Mostly found in breast-fed infants	Folate production in the intestines of infants mainly	[73,74]
*Bifidobacterium pseudocatenulatum*	Stomach	Intestine cancer prevention, Enhancement of host immune responses, maintain liver functionality	Milk, dairy products, and other carbohydrate source such as xylan or arabinoxylan	Modulate the gut–bone axis, inhibit inflammation, blocking Pro inflammatory Cytokines, Inhibiting TLR4/NF-κB Signaling	[75,76,77]
*Akkermansia muciniphila*	Resides in the mucus layer of the large intestine	Increasing mucus thickness and increasing gut barrier function	Cranberries, grapes, black tea, and walnuts	Protecting and strengthening your gut lining, prevents inflammation, manage glucose level in body	[78,79,80]
*Faecalibacterium prausnitzii*	Inside the intestine	High production of SCFAs that escape digestion and absorption in small intestine	Fruits and vegetables such as chicory roots, wheat, onion, banana, garlic, and leek	Weakened intestinal anti-inflammatory and immune regulation capabilities.	[81]
*Lactobacillus acidophilus*	Stomach, duodenum, and jejunum	Inhibiting carcinogen and mutagen formation, altering overall metabolism	Milk enriched with acidophilus, yoghurt, miso, and tempeh	Treat bacterial vaginosis, yeast infection, digestive disorder, and some neurological disorder	[82,83]
*Lactococcus lactis*	Passage of GI	Improved the growth performance and regulated amino acid profiles, intestinal immunity, and microbiota in weaning piglets	Yogurt, cheese, and sauerkraut	Exhibit protection against non-respiratory pathogens, such as HIV, Human papillomavirus and the malarial parasite	[84]
*Lactiplantibacillus plantarum*	Small intestine barrier	Immunomodulating properties and decrease the anti-inflammatory cytokine	Kimchi, Ogi, sourdough, and fermented plant material, and fermented sausages	Antioxidant, cancer-preventative, anti-inflammatory, antiproliferative, anti-obesity, and anti-diabetic properties	[85]
*Clostridium butyricum*	Intestinal tract	Intestinal microbiota disorder in human and enhance the humoral immune response	Soured milk and cheeses.	suppress inflammatory cytokine secretion, and modulate CNS autoimmunity, inhibit the increase in IL-17A gene expression	[86,87]
*E. coli*	Lower intestine	Keep digestive system healthy, breakdown and digestion of food	Raw vegetables and undercooked ground beef and contaminated water	Growth of tumor inhibit by ClyA toxin *(E. coli)*, decreased the postprandial blood glucose, antipyretic, anti-inflammatory, and anti-amyloidic	[88]

### 4.5. Recent Evidence for Probiotics and Neurodegenerative Disorders

In neurodegenerative disorders, neuroinflammation plays a very crucial role in pathogenesis, which has been proven through various studies. That may further drive the progressive loss of dopaminergic neurons. Therefore, increasing efforts on anti-inflammation approaches are being made in developing a cure for ND [89]. Different probiotics such as *E. coli*, *Lactiplantibacillus plantarum*, *Bifidobacterium pseudocatenulatum* and other combinations of probiotics capsules or tablets can useful as anti-inflammatory, anti-oxidant, or anti-pro inflammatory cytokines release and reduce the chances of occurring ND in patients [90]. Numerous neurological and psychiatric illnesses are linked to altered amounts of 5-HT (5-hydroxytryptamine-Serotonin) and DA (dopamine) to regulate this signaling molecule VSL#3 such as complex probiotic (consists of eight bacterial strains) VSL#3 interacts with mesenchymal stromal cells (hMSCs) to reduce neurodegeneration and inhibit NOD-like receptor protein-3, which mediated inflammation without altering the effects of hMSCs [91]. Cognitive processes, learning, memory, and emotional changes can all be modulated by NA. The two major inhibitory and excitatory chemicals are GABA and ACh. With respect to a single strain *Bifidobacterium longum* improve the cognitive function in healthy Balb/c mice, another context of using a multistrain probiotic, including different species of *Lactobacilli* and *Bifidobacterium* in the adult population, demonstrated improved cognition [92]. In accordance with the recent study to analyze the effect of a diet containing appropriate bacteria, participants were asked to consume two capsules after the meal in the morning and evening, which made a total of four capsules (a total of 1 × 10^9^ colony-forming a unit of *Bifidobacterium bifidum* BGN4 and *Bifidobacterium longum* BORI in soybean oil) to be taken per day for 12 days and then the relative abundance at genus level of *Clostridiales* and *Prevotellaceae* has been observed [93].

#### 4.5.1. Invertebrate System Studies

The beneficial effect of probiotics for neurodegenerative disease can be studied through an invertebrate model system, which is quite cheaper, and translational. Because of the complexity of the human nervous system and microbiota, identifying the primary microbial proteins or metabolites that have a direct influence on host neurons during neurodegeneration is usually difficult [94]. Simpler organisms like *C. elegans* were utilized in order to better understand the microbe-host interaction in the context of NDs. A recent study of the effect of probiotics on invertebrate models shows that, protein aggregation of α-synuclein, movement analysis, or locomotor analysis were restored with the help of a single bacterial strain (*Lactobacillus plantarum*) or multiple bacterial strains (*E. coli OP50* and *B. subtilis NCIB3610*) [95].

In the PD model (*C. elegans*), expression of α-synuclein was expressed in disease the condition [96]. The Caldwell lab used a genome-wide RNAi screen to identify the role of the endocytic pathway in reducing α-synuclein toxicity in order to identify genetic variables that influence α-synuclein-mediated proteotoxicity and treated *C. elegans* with *B. subtilis PB6* and *Bifidobacterium dentium* respectively that regulate the endocytic pathway through degradation of α-synuclein aggregates [97]. The PXN21 protein from *B. subtilis* prevents and prevents α-synuclein from aggregating [98]. The adult-onset loss of the dopaminergic neurons and locomotor dysfunction were induced by Ala-53-Thr, Ala-30-Pro, or Gln-46-Tyr mutations in the α-synuclein gene in the *D. melanogaster* variant of the UAS-Synuclein experiment, this locomotor dysfunction was restored by *B. subtilis PXN21* and *Lactobacillus plantarum* within 4–12 days [99].

In the *D. melanogaster* strains of UAS-BACE/UAS-APP, *L. brevis* and *Bifidobacterium dentium* are less prevalent in the gut, and GABA levels are decreased in the CNS [100]. Similarly for AD model, uncoordinated locomotion, the buildup of insoluble tau, and age-related neuronal loss and deterioration are all present in *C. elegans* (A53T) mutant [101]. Although, Tan et al. found that *Lactobacillus*, particularly the *L. plantarum DR7* strain, restores the rough eye phenotype in *D. melanogaster* GMR-A42 AD flies. The *L. plantarum* DR7 can restore the gut microbiota diversity in flies by increasing the abundance of *Stenotrophomonas* and *Acetobacter*, with reducing *Wolbachia* [102]. Although in other ND, like ALS; the generation of SOD1 (G85R) mutations in *C. elegans* led to severe locomotor defects and the formation of insoluble SOD1 aggregates in the perinuclear region of motor neurons [103]. In a demethylase-dependent manner, the KDM5 protein controls the immune deficiency (IMD) signaling pathway and maintains bacterial balance in *D. melanogaster* [104].

#### 4.5.2. Vertebrate System Studies

The prevalence of ND is on the increase worldwide as the population ages, posing a serious danger to human health. Probiotics, live microorganisms that help the host’s health, may hold promise in the treatment and prevention of these crippling diseases, according to a recent study. Vertebrate models have become an important resource in this context for understanding the fundamental processes of neurodegeneration and evaluating the effectiveness of probiotics in reversing it. With respect to AD, according to Webberley et al.’s 2022 research in 3xTg mice, the Lab4b probiotic acts as a neuroprotective agent through an anti-inflammatory cytokine, and it has also been demonstrated that IL-10 absence lessens disease pathology in AD animals [105]. Similarly, Yang et al. discuss the importance of Acidophillus-KAL4 in reducing gut barrier damage and inflammation in elderly SAMP8 mouse models, as well as lowering levels of LPS and γ-H2AX, 8-OHdG, TLR4, RIG-I, and NF-κβ nuclear translocation in the brain [106]. In respect to PD, Sun et al. created male C57BL/6 (MPTP initiated) mice, and they investigated whether reversing gut microbiome dysbiosis was possible and *Clostridium butyricum* therapy for four weeks resulted in reduced amounts of colonic GLP-1, colonic GPR41/43, and cerebral GLP-1 receptor in MPTP-induced rodents [107]. By shedding light on the underlying mechanisms of these conditions and testing the efficacy of probiotics in vertebrate models, we might develop treatments that fight against these terrible illnesses Table 3.

### 4.6. Mechanism of Action and Therapeutic Effect of Probiotics in Combatting Neurological Disorders

Probiotics, which have been shown to have health benefits when consumed, have drawn a lot of interest in recent years because of their ability to treat and avoid a variety of diseases. While the exact mechanism of action of probiotics is not yet fully understood, growing evidence suggests that they act through various pathways to regulate immune function, improve gut barrier function, modulate the gut-brain axis and neurological complications [134].

Based on the results of both the animal and human studies, the consumption of probiotics has a significant beneficial effect on AD Table 3. We can conclude that most of the study is based on *Bifidobacterium* and *Lactobacillus* and as an outcome this study reveals that probiotics can improve memory dysfunction and cognitive dysfunction in similar to neurodegenerative diseases [87,98]. With respect to AD, the CNS’s inflammatory reaction to damage or infection is called neuroinflammation, which is accompanied by an accumulation of glial cells. Activated microglia and astrocytes generate pro-inflammatory cytokines like IL6, IL8, and IL10, and these cytokines directly cause neuronal injury [135]. According to studies, probiotics can restore chronic inflammation, the function of clearing abnormal proteins, and synaptic dysfunction. Neurodegeneration and brain loss are caused by all of these occurrences. To understand and solve the puzzle of how probiotics were beneficial in AD, there have primarily been four clinical trials, which are mentioned in Table 4, below. Regarding PD, a new clinical trial of probiotic capsules (containing *Lactobacillus acidophilus*, *Bifidobacterium bifidum*, *Lactobacillus reuteri*, and *Lactobacillus fermentum*) demonstrates the same effects as the MDS-UPDRS [136]. Another 2019 research demonstrates that the major pro- and anti-inflammatory cytokines, as well as ROS, are produced by *Lactobacillus* and *Bifidobacterium* genus when peripheral blood mononuclear cells (PBMCs) isolated from people with Parkinson’s disease (PD) are compared to healthy participants [137]. In relation to other ND *Lacticaseibacillus rhamnosus* HA-114 can improve the energy balance and cholesterol homeostasis in ND animals [131]. In relation to neurodegenerative disease, only 10% of the research concentrates on *Streptococcus* and *Clostridium* species. So on, we give a summary of the literature in this systematic review by using data that was extracted from different sources.

### 4.7. Future Aims and Conclusions

The use of probiotics in neurodegenerative diseases such as Alzheimer’s, Parkinson’s, and other neurodegenerative disorders is an area of ongoing research, and there is not yet a definitive conclusion on its effectiveness. Some studies suggest that probiotics may have the potential in reducing inflammation and oxidative stress, improving gut microbiota composition, and enhancing cognitive function in neurodegenerative diseases. However, these findings are often based on invertebrate, animal models, or small-scale human studies, and more research is needed to confirm their efficacy and safety. It is important to note that probiotics should not be considered a cure or a standalone treatment for neurodegenerative diseases. They should be used as part of a comprehensive treatment plan, including medication, lifestyle changes, and other interventions recommended by healthcare professionals. Overall, while the use of *Bifidobacterium* and *Lactobacillus* in neurodegenerative diseases is a promising area of research, more large-scale, randomized controlled trials are needed to better understand their potential benefits and limitations.

## Figures and Tables

**Table 1:** Inclusion and exclusion criteria.

	Inclusion Criteria	Exclusion Criteria
Study design	Review articles and research studies that investigated probiotics and Neurodegenerative diseases	Case reports, Hypothesis, Communication, Systematic review and Current opinion
Population	Humans with no age restriction and any vertebrate or invertebrate model system studies	N/A

**Table 3:** Study of neurodegenerative diseases and their mediation with probiotics on different invertebrate and vertebrate model systems.

Model System	ND	Mediation	Outcome	Refs.
Study History in an Invertebrate Model System				
*C. elegans*	PD	*B. subtilis PXN21* *Lactobacillus plantarum*	Inhibits and reverses α-syn aggregation, improved locomotion and reduced dopaminergic neuron degeneration	[108]
	AD	*E. coli OP50* *B. subtilis NCIB3610*	Alleviated the paralysis phenotype, behavioral deficits, and aggravate lifespan	[109]
	ALS	*Lacticaseibacillus rhamnosus HA-114*	Suppresses the progression of motor neuron degeneration	[110]
	HD	*Bacillus subtilis*	Extends longevity through downregulation of the insulin-like signaling pathway	[111]
*D. melanogaster* Park^25^ flies	AD	*Lactobacillus plantarum DR7*	Ameliorate the AD effects	[112]
		*Bifidobacterium longum* ssp. *infantis NCIM 702 255*	Rescued amyloid beta deposition and acetylcholinesterase activity	[113]
		*Lactobacillus* spp.	Improved gene expression related to insulin signaling, fat metabolism	[114]
	PD	*Acetobacter tropicalis*	Reduced mitochondrial function and disrupted insulin-like signaling and glucose regulation	[115,116]
	ALS	*Lactobacillus plantarum*	Degeneration of motor nerve cells	[117]
	HD	*E. coli*	Regulating HTT aggregates and motor defects	[118]
**Study history in vertebrate model system**				
3 × Tg AD † mice	AD	*Lactobacillus plantarum PS128*	Regulated glycogen synthase kinase 3 beta activity	[119]
		*Bifidobacterium breve A1*	Reduce the expression of Aβ gene	[120]
		*SLAB51*	It degrade Aβ plaque through CathepsinD	[121]
5× FAD Tg mice		*L. plantarum C29* *Bifidobacterium lactis CUL 34*	Decrease the expression of Caspase 3 and increase the activation of microglial	[122]
Wistar rats		*L. plantarum MTCC 1325*	Increase the concentration of ACh in hippocampus	[123]
Patient (Infant aged < 7 months)		*B. breve M-16V*	No effect on AD marker	[124]
Pregnant woman		*B. longum BB536*	Prevent eczema and AD in their offspring	[125]
Children aged (1–3 years)		*L. fermentum* *L. plantarum CJLP133*	Decrease IFNγ and IL4	[126]
Adult patients		*B. animalis LKM512*	Reduce the cumulative incidence of AD	[127]
MitoPark PD † mice	PD	*Lactobacillus rhamnosus GG* *Bifidobacterium bifidum*	Decrease the level of dopaminergic neuronal loss	[128]
C57BL/6 mice MPTP † -induced PD		*Streptococcus thermophilus CRL808*	Increase pro-inflammatory receptors concentration (IL10)	
		*Bifidobacterium bifidum* *Lactobacillus reutri* *Lactobacillus fermentum*	Increase PPARγ	
6-OHDA † induced PD † in C57BL/6 mice		*SLAB51*: *Streptococcus thermophilus*, *Bifidobacterium longum*, *Bifidobacterium breve*, *Bifidobacterium infantis*, *Lactobacillus acidophilus*, *Lactobacillus plantarum*, *Lactobacillus paracasei*, *Lactobacillus delbrueckii* sub sp. *bulgaricus*, *Lactobacillus brevis*	Induces hippocampal long-term potentiation through BDNF	[129]
PD patients		*Streptococcus salivarius* sub sp. *thermophilus*, *Enterococcus faecium*, *Lactobacillus rhamnosus GG*, *Lactobacillus acidophilus*	Decrease IL1, IL8 and TNFα	[130]
		*Bifidobacterium infantis*	Reduce abdominal pain and constipation	
Mice	ALS	*Lacticaseibacillus rhamnosus HA-114*	Through mitochondrial β-oxidation restores lipid equilibrium and energy balance	[131]
C57BL/6 mice	HD	*Bifidobacterium* spp.	decreased *Il-1β* and *Il-6* and increased *TGF-β* and *Il-10* expression	[132]
BALB/c mice		*L. casei IMV B-7280*	Inhibiting NF-κB pathway	[133]
C57/BL6 mice		*L. lactis NZ9000SHD5*	reduced colonic glandular structure, downregulated expression of inflammatory molecule	[132]

(† = Disease induced mice model).

**Table 4:** Recent clinical studies of ND with combination or singular probiotic with their therapeutic effect and mechanism of action.

ND	Probiotics	Duration	Mechanism	Therapeutic Effect	Ref.
AD	*L. acidophilus* *L. cases* *B. bifidum* *L. fermentum*	12 weeks	Management of metabolic deviation	Decreased level of triglyceride in blood Decreased serum malondialdehyde Unproductive on antioxidant capacity.	[138]
	*L. fermentum* *L. plantarum* *B. lactis* *L. acidophilus* *B. bifidum* *B. longum*	12 weeks	Management of Urea and Glucose level	Improved cognitive function. Unproductive on antioxidant capacity. Improved level of Glutathione Reduced level of Deoxyguanosine.	[139]
	*L. acidophilus* *B. bifidum* *B. longum* selenium	12 weeks	Metabolic deviation and oxidative stress balancing	Decreased level of triglyceride in blood Improved the level of Glutathione Improved antioxidant capacity.	[140]
	*L. casei W56* *L. lactis W19* *L. acidophilus W22* *B. lactis W52* *L. paracasei W20* *L. plantarum W62* *B. lactis W51* *B. bifidum W23* *L salivarius W24*	28 days	Simulating the microbiota gut brain axis and active immune cells	Improved *Faecalibacterium* concentration in faecal. High concentration of kynurenine in blood. Improved RNA concentration in faecal bacteria.	[141]
PD	*Blautia* *Roseburia*, *Coprococcus* *Firmicutes* *Proteobacteria* *Verrucomicrobia*, *Oscillospira* *Bacteroides*	NA	Maintaining level of fecal microbiota and mucosal level	Pro inflammatory dysbiosis, trigger inflammation Induced misfolding of α synuclein.	[142]
	*B. bifidum*	NA	Regulation of neuronal inflammation	Reduced neuroinflammation.	[143]
	*L. salivarius* *L. plantarum* *L. acidophilus* *L. rhamnosus* *B. animalis* subsp. *B. breve*	NA-	Monitoring the production of cytokine, superoxide anions (O_2_-); Proliferation of *E. coli* and *K. pneumoniae* DNA for tyrosine decarboxylase	Reduced ROS generation, improved constipation, and inhibition of E. coli and K. pneumonia all contribute to anti-inflammatory activity.	[144]
Multiple Sclerosis	*B. animalis*	NA	Regulation of gut barrier permeability	Improved gut barrier function and reduced inflammation.	[145]
ALS	*L. rhamnosus*	NA	Regulation of behavioral changes	Improved behavior and social interaction.	[146]
	*B. longum*	NA	Regulation of stress and anxiety level	Reduced anxiety and depressive symptoms.	[147]

## Data Availability

Not Applicable.
